# Genetic Diversity of EBV-Encoded LMP1 in the Swiss HIV Cohort Study and Implication for NF-Κb Activation

**DOI:** 10.1371/journal.pone.0032168

**Published:** 2012-02-22

**Authors:** Emilie Zuercher, Christophe Butticaz, Josiane Wyniger, Raquel Martinez, Manuel Battegay, Emmanuelle Boffi El Amari, Thanh Dang, Jean-François Egger, Jan Fehr, Esther Mueller-Garamvögyi, Andrea Parini, Stephan C. Schaefer, Franziska Schoeni-Affolter, Christine Thurnheer, Marianne Tinguely, Amalio Telenti, Sylvia Rothenberger, and the Swiss HIV cohort study

**Affiliations:** 1 Institute of Microbiology, University Hospital and University of Lausanne, Lausanne, Switzerland; 2 Division of Infectious Diseases and Hospital Epidemiology, University Hospital Basel, Basel, Switzerland; 3 University Clinic of Infectious Diseases, University Hospital of Geneva, Geneva, Switzerland; 4 Infectious Diseases Service, University Hospital and University of Lausanne, Lausanne, Switzerland; 5 Pathology, Laboratoire Viollier Weintraub SA, Geneva, Switzerland; 6 Division of Infectious Diseases and Hospital Epidemiology, University Hospital and University of Zurich, Zurich, Switzerland; 7 Institute of Pathology, University Hospital and University of Bern, Bern, Switzerland; 8 Medical Service, Ospedale Regionale, Lugano, Switzerland; 9 Institute of Pathology, University Hospital and University of Lausanne, Lausanne, Switzerland; 10 Swiss HIV Cohort Study Data Center, University Hospital and University of Lausanne, Lausanne, Switzerland; 11 Division of Infectious Diseases, University Hospital and University of Bern, Bern, Switzerland; 12 Institute of Surgical Pathology, University Hospital and University of Zurich, Zurich, Switzerland; The Chinese University of Hong Kong, Hong Kong

## Abstract

Epstein-Barr virus (EBV) is associated with several types of cancers including Hodgkin's lymphoma (HL) and nasopharyngeal carcinoma (NPC). EBV-encoded latent membrane protein 1 (LMP1), a multifunctional oncoprotein, is a powerful activator of the transcription factor NF-κB, a property that is essential for EBV-transformed lymphoblastoid cell survival. Previous studies reported LMP1 sequence variations and induction of higher NF-κB activation levels compared to the prototype B95-8 LMP1 by some variants. Here we used biopsies of EBV-associated cancers and blood of individuals included in the Swiss HIV Cohort Study (SHCS) to analyze LMP1 genetic diversity and impact of sequence variations on LMP1-mediated NF-κB activation potential. We found that a number of variants mediate higher NF-κB activation levels when compared to B95-8 LMP1 and mapped three single polymorphisms responsible for this phenotype: F106Y, I124V and F144I. F106Y was present in all LMP1 isolated in this study and its effect was variant dependent, suggesting that it was modulated by other polymorphisms. The two polymorphisms I124V and F144I were present in distinct phylogenetic groups and were linked with other specific polymorphisms nearby, I152L and D150A/L151I, respectively. The two sets of polymorphisms, I124V/I152L and F144I/D150A/L151I, which were markers of increased NF-κB activation *in vitro*, were not associated with EBV-associated HL in the SHCS. Taken together these results highlighted the importance of single polymorphisms for the modulation of LMP1 signaling activity and demonstrated that several groups of LMP1 variants, through distinct mutational paths, mediated enhanced NF-κB activation levels compared to B95-8 LMP1.

## Introduction

Epstein-Barr virus (EBV) infects more than 90% of human adults worldwide. EBV causes infectious mononucleosis and is associated with several human malignancies, among them nasopharyngeal carcinoma (NPC), Burkitt's lymphoma and Hodgkin's lymphoma (HL) [1]. EBV infection of B-lymphocytes is mostly non-lytic and results in the expression of a limited number of nuclear and membrane proteins. EBV-encoded latent membrane protein 1 (LMP1) is a multifunctional oncoprotein essential for EBV-induced B-cell proliferation and transformation in vitro [2], [3], [4], [5]. LMP1 also has transforming effects on non-lymphoid cells such as rodent fibroblasts and keratinocytes [5], [6], [7]. LMP1 is a powerful inducer of nuclear factor-κB (NF-κB)-mediated transcription [8], [9], a property that is essential for EBV-transformed lymphoblastoid cell survival [10]. NF-κB plays a determinant role in cell transformation: tumor promotion is mediated by its anti-apoptotic functions and aberrant activation of NF-κB is associated with tumorigenesis [11], [12]. Moreover, this transcription factor is essential for the progression of EBV-associated lymphomas in vivo [13]. Up to date, the vast majority of functional studies on LMP1 have used as a prototype B95-8, an infectious mononucleosis derived isolate. CAO LMP1, a variant isolated from a NPC, was found to have increased potential to transform rodent fibroblasts and to induce tumors in nude mice, when compared to B95-8 LMP1 [14], leading to the hypothesis that polymorphisms within LMP1 gene influences the susceptibility to develop EBV-associated tumors. When compared to B95-8 LMP1, CAO LMP1 induces higher levels of NF-κB activation [15]. This observation has been extended to several cell types, such as HEK 293, Elijah-BL, Daudi BL, DG75 and Jurkat [16], demonstrating that this property is not cell-type specific. However, the exact polymorphisms allowing increased NF-κB activation levels had not been mapped to date.

Patients infected with human immunodeficiency virus (HIV) are at high risk of developing EBV-associated lymphoproliferative disorders [17]. Earlier, a study of the Swiss HIV Cohort demonstrated that immune responses contribute to the development of EBV-associated brain lymphoma [18]. However, the contribution of viral factors to the development of EBV-associated malignancies is not well understood. EBV has been detected in 80–100% of Hodgkin's lymphoma arising in the setting of HIV, supporting the notion that EBV plays a pivotal role in the pathogenesis of this type of tumor [19], [20]. Since the introduction of highly active antiretroviral therapy (HAART) the total number of cancer cases has decreased in HIV-infected individuals worldwide. In contrast, the frequency of HL among HAART treated HIV-infected patients increases with the prolonged life expectancy [19], [21], [22], suggesting that HIV-induced immunosuppression is not the unique factor involved in the etiology of HL and that other factors such as genetic viral variations could play a role in the development of this kind of tumor. Of special importance is the detection of EBV type II latency program in HL, which is characterized by the expression of LMP1 and two other viral proteins [23]. The strong association between EBV and HL in HIV-infected individuals (HIV-HL) and the limited number of viral products expressed in the tumor cells provide ideal conditions to study the impact of LMP1 genetic variations on the etiology of EBV-associated tumors.

We mapped several single amino acid polymorphisms leading to significantly enhanced capacity for NF-κB activation compared to B95-8 LMP1 and used samples from the Swiss HIV Cohort Study (SHCS) to evaluate the importance of LMP1 polymorphisms relevant to NF-κB activation in the etiology of EBV-associated HL.

## Materials and Methods

### Samples

Paraffin blocks of biopsies of B-cell and T-cell lymphomas were provided by the Institute of Pathology in Lausanne. One biopsy from a patient with a post-transplant lymphoproliferative disorder (PTLD) was obtained from Geneva University Hospital. Biopsies specimen of Hodgkin's lymphoma of HIV-infected individuals were obtained from the Institutes of Pathology of the universities of Bern, Geneva, Lausanne and Zurich. Blood samples of HIV-infected individual were provided by several centers of the SHCS: Bern, Basel, Geneva, Lausanne, Lugano and Zurich. This study was approved by the scientific review board of the Swiss HIV Cohort Study. All SHCS participants provided informed written consent and the SHCS has been approved for Lausanne by the commission cantonale d'éthique de la recherche sur l'être humain du canton de Vaud.

### DNA extraction

DNA of tumor samples of B and T cell lymphomas and a case of PTLD was prepared according to standard procedures in pathology centers of Lausanne and Geneva. DNA of tumor samples from Zurich was extracted at the pathology center of Zurich according to standard procedures. Isolation of DNA from biopsies and cell samples of HIV-infected individual was performed with QIAamp DNA Mini Kit (QIAGEN, Basel, Switzerland) according to manufacturer's instructions. Frozen biopsies were crushed before DNA extraction. Paraffin from paraffin embedded biopsies was removed by dissolution with xylol 100% before DNA extraction.

### Amplification by polymerase chain reaction and isolation of LMP1 genes

The region between positions 169.508 and 168.111 of the EBV genome was amplified using a specific primer pair based on the published prototype B95-8 LMP1 sequence [24]. PCRs were carried out either with Pwo DNA Polymerase (Roche Applied Science, Rotkreuz, Switzerland) on the four biopsies of EBV-associated tumors (A1, A2, A3 and P1) or with AmpliTaq Gold DNA Polymerase (Applied biosystems) on samples from HIV-infected individuals. Primers and PCR programs are available in Materials and Methods S1. PCR products were purified with QIAquick Gel Extraction Kit (QIAGEN) or MSB Spin PCRapace (Invitek, Berlin, Germany) and sequenced directly using ABI PRISM BigDye Terminator Cycle Sequencing Ready Reaction Kit (Applied Biosystems, Foster City, CA, USA). The whole gene was subcloned into a eukaryotic expression vector, pCR3.1 (Invitrogen, Basel, Switzerland), using adapters with HindIII and XbaI restriction sites. At least two independent clones of each variant were sequenced on both strands. B95-8 and AG876 full length LMP1 were constructed by cloning LMP1 gene from the lymphoblastoid cell lines B95-8 [25] and AG876 (kindly provided by A. Rickinson, Birmingham, UK) into pCR3.1 vector. CAO LMP1 (GeneBank AF304432, kindly provided by F. Grässer, Homburg, Germany) was subcloned into the same background vector. LMP1 constructs were cloned from genomic DNA samples.

### Construction of LMP1 mutants

Chimeras were built by enzymatic digestion of the gene encoding B95-8, A2 and P1 LMP1 using the internal DNA restriction sites NaeI or BglII. Mutations were introduced by PCR using Pwo polymerase. All constructs were cloned into the expression vector pCR3.1 and sequenced. Plasmids were amplified and purified using Qiagen plasmid MIDI kit (QIAGEN) and quantified using NanoDrop ND 1000 Spectrophotometer (NanoDrop Technologies, Wilmington, DE, USA).

### Cell culture

Human embryonic kidney 293 (HEK) [26] and HEK 293T (ATCC: CRL-11268) cells were cultured in Dulbecco's modified Eagle medium (DMEM - Gibco, Basel, Switzerland) supplemented with 10% (v/v) heat-inactivated fetal bovine serum, penicillin and streptomycin at 37°C with 5% CO_2_. Lymphoblastoid cell lines B95-8 and AG876 were cultured in Roswell Park Memorial Institute medium (RPMI - Gibco) supplemented with 10% (v/v) heat-inactivated FCS, penicillin and streptomycin at 37°C with 5% CO_2_.

### Gene reporter assay

Gene reporter assay system was used to measure NF-κB activation levels induced by LMP1. Expression vectors for either prototype B95-8 LMP1, variants or mutants were co-transfected with the reporter κB-conA-luc (kindly provided by F. Grässer) that comprises *Firefly* luciferase gene under the control of a conalbumin reporter with 3 integrated κB elements derived from the immunoglobulin κ chain enhancer. HEK cells were transfected in 24-well plates with 50 ng of NF-κB *Firefly* reporter construct κB-conA-luc and 50 ng of LMP1 expression vectors using FuGENE 6 (Roche Applied Science). Twenty-four hours after transfection, cells were lysed in Cell Culture Lysis Reagent (Promega, Amriswil, Switzerland) and assayed for light emission in a tube luminometer Lumat LB 9507 or a microplate luminometer Tristar LB 941 (BERTHOLD TECHNOLOGIES, Bad Wildbad, Germany) using Luciferase Assay System (Promega). Final quantifications were calculated on three separate experiments performed in triplicates. Statistical analyses were performed using GraphPad Prism version 5.04 for Windows (GraphPad Software, http://www.graphpad.com).

### Immunoblot

Samples from gene reporter assays were boiled at 95°C for 4 min in 2× sample buffer (62.5 mM Tris-HCl pH 6.8, 20% glycerol, 2% SDS, 100 mM DTT) and separated through 10% SDS-polyacrylamide gel electrophoresis, then transferred to nitrocellulose membrane, probed with the appropriate antibodies and revealed by enhanced chemiluminescence (ECL - LiteAblot, Euroclone, Italy). Primary antibodies were used at the following dilutions: LMP1 rat monoclonal 8G3 [27], 1∶500–1∶1.000 (provided by F. Grässer). Mouse anti-alpha-tubulin (Sigma, Buchs, Switzerland), 1∶10.000. Secondary antibodies were used at the following dilutions: Polyclonal rabbit anti-mouse HRP and polyclonal rabbit anti-rat HRP (Dako Cytomation, Glostrup, Denmark), 1∶4.000–1∶5.000.

### Cell viability

Toxicity of the different LMP1s was measured using CellTiter-Glo® Luminescent Cell Viability Assay (Promega) according to manufacturer's protocol. This method allows determining the amount of viable cells by measuring ATP levels in samples. Briefly, HEK cells in 24-well plates were transfected with 50 ng expression plasmids using FuGENE 6. Fourteen hours post transfection cells were seed in 96-well plates and twenty-four hours post transfection cells were lysed in CellTiter-Glo® Reagent and assessed for luminescent signal in a microplate luminometer Tristar LB 941.

### Phylogenetic tree

For phylogenetic analysis, LMP1 nucleotide sequences from position 169474 to 168160 of EBV genome were aligned with Geneious (v5.1; A. J. Drummond et al., Biomatters Ltd., http://www.geneious.com) and controlled manually for repeats and deletion alignment. The alignment was used to build neighbor-joining tree (Jukes-Cantor model) using Geneious.

### EBV typing

EBV type was determined on EBNA2 gene as described in Telenti *et al.* [28]. The method is fully described in Materials and Methods S1.

### Analysis of samples from patients of the SHCS with or without HL

Among participants of the SHCS, 48 have had a confirmed diagnosis of HL at the date of our study. We included in our study 42 HIV-HL with available blood samples (37 men and 5 women; age at the date of blood sampling 44.2±10.7 years) and 90 SHCS participants without any tumor history (72 men and 18 women; age at the date of blood sampling 41.9±9.2 years). For 16 HIV-HL we obtained blood as well as tumor biopsy. Sequences of LMP1 gene encoding amino acids 96 to 202 were determined using PCR amplification on genomic DNA with AmpliTaq Gold DNA polymerase followed by sequencing with nested primers, using the same method as above. Sequences were obtained for 41/42 HIV-HL (36 men and 5 women, mean age 44.3±10.8 years), for 15/16 biopsies and for 85/90 participants without tumor (67 men and 18 women, mean age 42±9.4 years). The strength of the association between the two sets of polymorphisms I124V/I152L and F144I/I150A/L151I and the etiology of HL was tested with a simple logistic regression and then with a logistic regression model controlling for sex, age, CD4 levels and the number of years of antiretroviral therapy at the date of blood sampling, using Stata 11 software (StataCorp, http://www.stata.com).

## Results

### Polymorphism F144I modulates NF-κB activation mediated by variant A2

**Figure 1:**
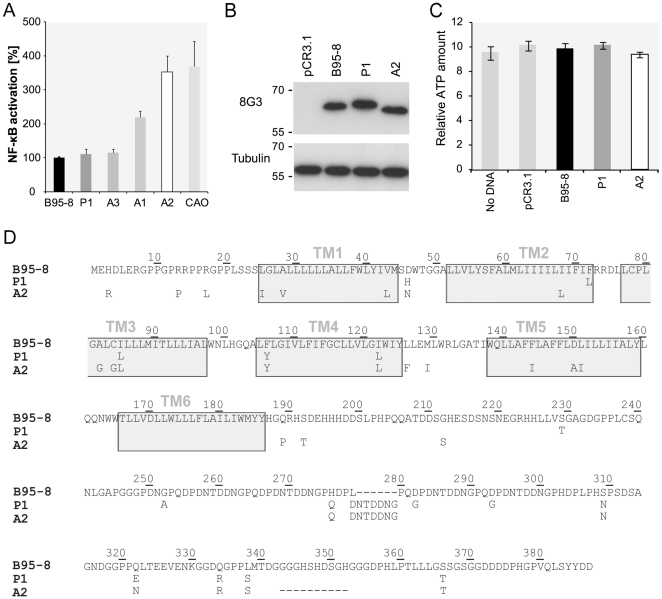
Analysis of LMP1 variants. (A) NF-κB activation by LMP1 variants. 293T cells were transfected with 50 ng of expression vector coding for LMP1 prototype (B95-8) and variants (P1, A3, A1, A2, CAO) and 50 ng of *Firefly* luciferase reporter plasmid. An analogous strategy of subcloning was applied to variants, prototype and CAO LMP1, to ensure appropriate comparisons. NF-κB activity was measured twenty hours after transfection using luciferase assay (Promega). Data are mean ± SD of triplicates and shown is a representative of three independent experiments with similar results. (B) Expression of LMP1 B95-8, A2 and P1 was visualized by SDS-PAGE and Western blotting with anti-LMP1 8G3 antibody. Detection against tubulin was used as internal control. (C) Measure of the toxicity of LMP1 variants. Cells were transfected with 50 ng of LMP1 B95-8, P1 or A2. Untransfected cells and cells transfected with empty vector were used as experimental controls. ATP amount was measured 24 hours after transfection using CellTiter-Glo Luminescent Cell Viability Assay (Promega). Shown is a representative experiment of three independent experiments with similar results. (D) Amino acid sequence alignment of B95-8, P1 and A2 LMP1. Only amino acids that differ from the sequence of prototype B95-8 LMP1 are indicated. Transmembrane segments are indicated by light gray boxes and deletions by dashes.

Among four LMP1 variants with different abilities to activate NF-κB we selected two variants, P1 and A2, on the basis of their activation profile to perform further studies (Figure 1A). The level of activation of variant P1 is close to that of prototype B95-8 LMP1, whereas variant A2 has a highly increased level of activation close to that of CAO LMP1. We observed that the prototype LMP1 and the two variants were all well expressed (Figure 1B) and non-toxic using a cell viability assay (Figure 1C). Furthermore enhanced NF-κB activation is not associated with a prolonged half-life, since half-lives of 3.6±0.6 and 2.8±0.4 hours were determined for P1 and A2, respectively (Figure S1). An overview of amino acid changes identified in variants P1 and A2 with respect to reference B95-8 LMP1 [24] is shown in Figure 1D. Variant A2 has a very high overall similarity to CAO LMP1 (GeneBank AF304432) [14], [15], [29]. A2 and CAO are highly polymorphic with 26 and 27 amino acid changes compared to B95-8 LMP1, respectively. Four polymorphisms L29V, V43L, D46N and I68L are unique to A2 and three, I137L, L178M and E328A, are unique to CAO, which also contains two additional direct repeats (Figure S2).

**Figure 2:**
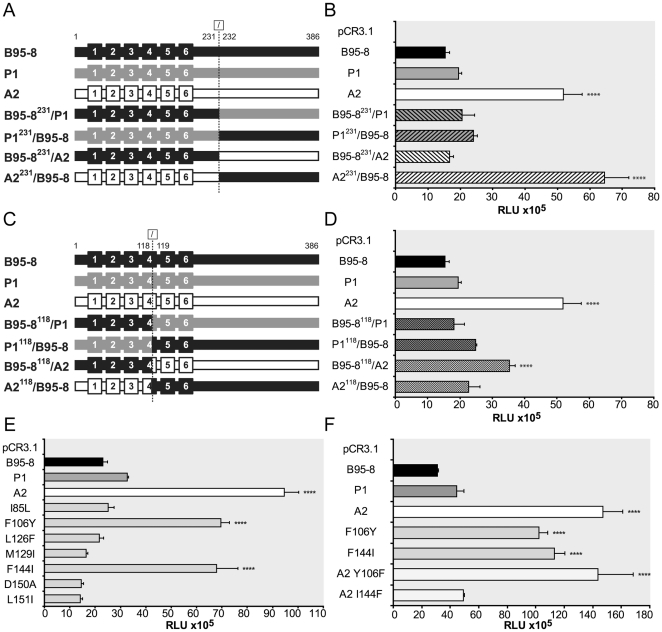
NF-κB activation levels by LMP1 chimeras and mutants. (A, C) Schematic representation of LMP1 chimeras split at amino acid 231 (A) and 118 (C). The six transmembrane segments are represented by boxes. (B, D–F) NF-κB activation by LMP1 chimeras split at amino acid 231 (B) and 118 (D), and LMP1 mutants on B95-8 background (E) and on A2 background (F). HEK cells were transfected with 50 ng of LMP1 vector and 50 ng of NF-κB reporter plasmid. Empty vector was used as control. NF-κB activity was measured twenty-four hours after transfection using luciferase assay (Promega). Shown are representative of three independent experiments with similar results. Data are given as mean ± SD of triplicates. Statistical analysis was done using one-way ANOVA with Bonferroni posttest using GraphPad Prism, n = 9 triplicates of three experiments. **** *P*<0.0001 relatively to the NF-κB activation of B95-8 LMP1. RLU: relative light units.

We then mapped polymorphisms responsible for the differences in NF-κB activation. In order to identify the regions implicated we constructed chimeras between variants and B95-8 LMP1. In a first set of chimeras, we used a NaeI restriction site to exchange the first 231 amino acids of either variant with B95-8 LMP1 and constructed the reciprocal chimeras using the same strategy (Figure 2A). Functional analysis by gene reporter assay showed that B95-8^231^/P1 and P1^231^/B95-8 activate NF-κB at levels close to P1 (Figure 2B). In contrast, B95-8^231^/A2 and A2^231^/B95-8 show a striking difference in their signaling potential. Whereas B95-8^231^/A2 induces NF-κB to level close to those of the prototype, A2^231^/B95-8 induces very high level of NF-κB activation, close to that of A2. Our results showed that polymorphisms within the 231 N-terminal amino acids of A2 are responsible for the increased capacity to activate NF-κB, whilst polymorphisms between amino acids 232 and 386, such as the 10 amino acids deletion and variations in the direct repeats, do not contribute to the enhanced NF-κB activation phenotype. To better refine regions associated with increased NF-κB activation, we exchanged the first 118 amino acids of either variant with B95-8 LMP1 using the naturally occuring BglII restriction site, and generated reciprocal constructs using the same strategy (Figure 2C). P1^118^/B95-8 is slightly more effective than the reciprocal construct B95-8^118^/P1 (Figure 2D). Neither B95-8^118^/A2 nor A2^118^/B95-8 displays the full A2 phenotype. Interestingly, A2^118^/B95-8 induces levels comparable to P1^118^/B95-8, suggesting that A2 and P1 share polymorphisms in the N-terminal part of the molecule (amino acids 1–118) associated with a modest increase in NF-κB activation levels with respect to the prototype. NF-κB activation level induced by B95-8^118^/A2 is significantly higher than that of the prototype, indicating that one or a combination of polymorphisms localized between amino acids 119 and 231 are associated with enhanced NF-κB activation. To prove this hypothesis we constructed chimeras by exchanging the amino acid 119–231 between variants and B95-8 LMP1 (Figure S3). The comparison of the amino acid sequences of variants P1 and A2 within residues 1 to 118 shows two common polymorphisms: I85L and F106Y (Figure 1D), which were introduced individually in the B95-8 sequence to evaluate their influence. Mutation F106Y, but not I85L, is associated with an increase in NF-κB activation level (Figure 2E and Table S1). Interestingly, the single mutation F106Y in the context of B95-8 increases NF-κB activation level 3-fold, whereas in the context of chimeras P1^118^/B95-8 and A2^118^/B95-8 its effect is limited indicating that other polymorphisms located between amino acids 1 and 118 counteract this effect. We tested the influence of 8 additional polymorphisms present in variant A2 between residues 119 and 231 (Figure 1D). Functional analysis of LMP1 mutants shows that mutation F144I leads to a significant increase in NF-κB activation (Figure 2E and Table S1). In contrast, other mutations are slightly deleterious (M129I, D150A or L151I) or neutral (Q189P, S192T and G212S). In order to confirm the importance of F144I we mutated isoleucine 144 to phenylalanine in variant A2. NF-κB activation level induced by A2 I144F is close to that of B95-8 (Figure 2F and Table S1). In contrast, mutation of tyrosine 106 to phenylalanine in variant A2 does not change NF-κB signaling potential of the variant, which was likely to be due to other polymorphisms regulating this effect. These results demonstrate that F144I is the unique polymorphism responsible for the high NF-κB activation level mediated by A2.

### Enhanced LMP1-mediated NF-κB activation is linked to polymorphisms in the transmembrane region

**Figure 3:**
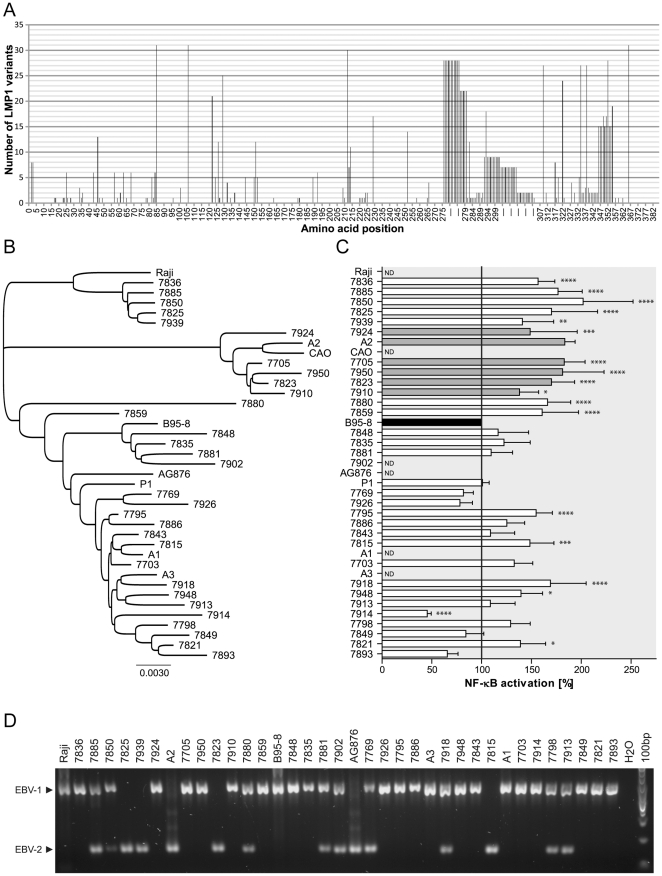
Analysis of LMP1 variants amplified from blood of HIV-infected individuals. (A) Frequency of polymorphisms from 31 LMP1 variants compared to the B95-8 prototype reference sequence. Dashes represent insertion of amino acids in direct repeats region compared to B95-8. Deletion of 10 (aa 343 to 352) or 23 (aa 332 to 354) amino acids are present in 13 and 2 variants, respectively. (B) Phylogenetic tree was built on LMP1 nucleotide sequences by employing neighbor-joining method using Geneious software. (C) Percentages of NF-κB activation induced by the 31 LMP1 variants. HEK cells were transfected with 50 ng of LMP1 vector and 50 ng of NF-κB reporter plasmid. NF-κB activity was measured twenty four hours after transfection using luciferase assay (Promega). Values were normalized to the B95-8 activation value fixed at 100%. Data are given as mean ± SD of percentages of triplicates of three independent experiments. Statistical analysis was done using one-way ANOVA with Bonferroni posttest using GraphPad Prism. * *P*<0.05, ** *P*<0.01, *** *P*<0.001, **** *P*<0.0001 relatively to the NF-κB activation of B95-8 LMP1. RLU: relative light units. ND: not done. (D) Typing based on EBNA-2 gene was performed on genomic DNA according to Telenti and coworkers [28]. B95-8 and Raji are EBV type 1 references and AG876 is EBV type 2 reference.

To better understand the importance of polymorphism F144I in the context of LMP1 sequence variation in individuals without a diagnosis of EBV-associated tumor we characterized LMP1 from blood of HIV-infected individuals included in the SHCS. Among randomly chosen genomic DNA, we obtained 31 full length LMP1 genes in a single amplification. The frequencies of amino acid changes compared to the B95-8 prototype are reported in Figure 3A. F106Y is present in 31/31 variants. The distribution of F144I is more restricted (5/31). 14 variants with a 10 amino acids deletion and 2 with a 23 amino acids deletion were isolated. A neighbor joining tree was built based on LMP1 nucleotide sequences from position 169474 to 168160 of EBV genome. Variants A1, A2, A3 and P1, the 31 variants isolated from HIV-infected individuals and the references LMP1 B95-8, CAO, Raji and AG876 were included in the analysis (Figure 3B and Figure S2 for alignment). Three different phylogenetic groups emerged from the tree. The first group comprising 5 LMP1 variants – 7836, 7885, 7850, 7825 and 7939– is characterized by two polymorphisms I124V and I152L, which always segregate together. The second group includes A2, CAO and the five variants 7924, 7705, 7950, 7823 and 7910. All variants from the second group are characterized by F144I, D150A and L151I and are more closely affiliated to the China1 strain described by Edwards et al. than to any other LMP1 strain [29]. The other variants formed a more heterogeneous group. Functional analysis revealed that a number of LMP1 variants display enhanced NF-κB activation profiles when compared to B95-8 LMP1 (Figure 3C). Variants from the first and second groups have all significantly increased capability to activate NF-κB compared to B95-8 LMP1. A few variants from the third group also display this phenotype. By sequence comparisons and directed mutagenesis we identified I124V as the amino acid change responsible for enhanced NF-κB activation of a variant of the first group, 7825 (Figure S4A). As shown above, F144 I is the determinant of the second group of variants. Finally, we found that F106Y leads to increased NF-κB activation in the context of variants 7795, 7815, 7918, 7948 and 7821 of the third group (Figure S4B). Since we have previously shown that this polymorphism has no influence in the context of A2 LMP1 (Figure 2F) and is present in all LMP1 variants sequences obtained in this study (Figure 1D and 3A), the effect of F106Y is modulated by sequence variations. To determine whether the phylogenetic groups are associated with an EBV subtype, we performed an amplification analysis based on the variability in EBNA-2 as described by Telenti and coworkers [28]. Twenty samples contained only EBV type 1 and had the same pattern as reference strains B95-8 and Raji; five samples contained only EBV type 2 as the reference strain AG876, and 9 samples harbored both EBV types (Figure 3D). The analysis showed that both EBV types are distributed among the phylogenetic groups and that some samples contained several EBV strains since both types were detected simultaneously. In summary, we found two phylogenetic groups of LMP1 in which all variants have increased ability to activate NF-κB when compared to B95-8 LMP1 and described I124V for the first group and F144I for the second group as polymorphisms involved in the increased NF-κB activation. Polymorphisms I124V/I152L and F144I/D150A/L151I always segregating together were used to represent the two phylogenetic groups, respectively.

### Polymorphisms I124V/I152L and F144I/D150A/L151I are not associated with HIV-HL

**Figure 4:**
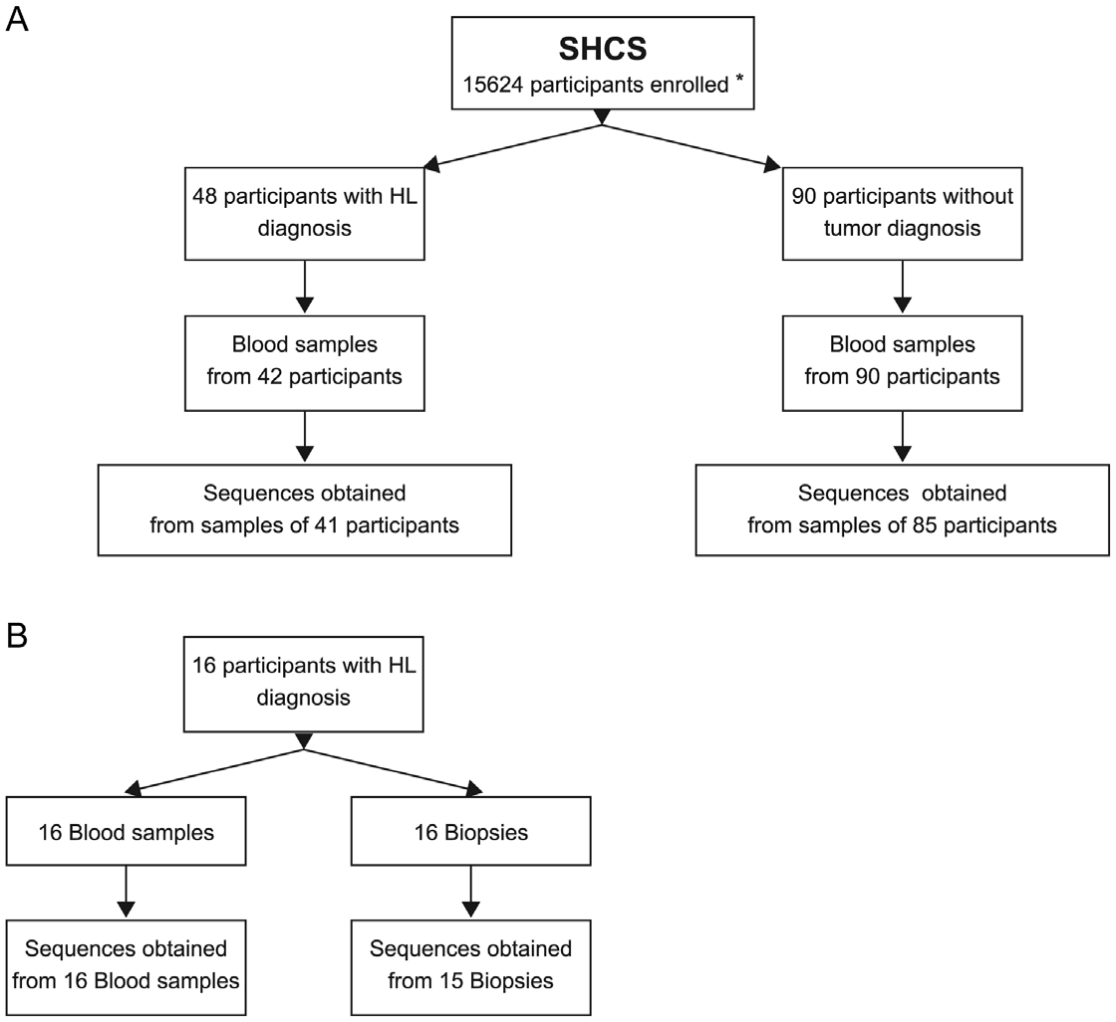
Flow chart of the study. (A) Comparison of LMP1 polymorphisms in blood samples from HIV-infected individuals with or without HL. * patients enrolled in the SHCS at the beginning of the study by March 2009 [60]. (B) Comparison of LMP1 polymorphisms in blood and corresponding biopsy from HIV-HL.

Since LMP1 driven NF-κB activation is essential for EBV-transformed cells survival, we tested whether enhanced NF-κB activation potential favors the etiology of EBV-associated HL. We analyzed samples from HIV-infected individuals enrolled in the SHCS who have had a diagnosis of HL or not in a pilot epidemiological study (Figure 4A). At the time of our study forty-eight HL were diagnosed among participants of the SHCS and blood samples from 42 participants were available. Blood samples from 90 participants without tumor diagnosis were used as comparators. To determine the presence of the relevant polymorphisms we amplified and sequenced a region of LMP1 gene corresponding to amino acids 96 to 202 (Figure 4A and Materials and Methods). Among the 41 HIV-HL with LMP1 amplification and sequence we detected single sequences in samples from 31 participants and two sequences in samples from 10 participants. Among the 85 participants without tumor diagnosis with LMP1 amplification and sequence we detected single sequences in samples from 36 participants, two sequences in samples from 46 participants and three sequences in samples from 3 participants.

We assessed the presence of polymorphisms I124V/I152L and F144I/D150A/L150I for each participant's sample (Table 1). I124V/I152L was found in 12.2% of HIV-HL and in 24.7% of participants without tumor. F144I/D150A/L150I was found in 41.5% of HIV-HL and in 31.8% of participants without tumor. The simultaneous presence of both sets of polymorphisms was found in blood from one HIV-HL and from four participants without tumor. The strength of the association between the polymorphisms of interest and the etiology of HL was tested with a simple logistic regression and then with a logistic regression model adjusted for sex, age, CD4 levels and the number of years of antiretroviral therapy at the date of blood sampling (Table 2). Our results showed that I124V/I152L and F144I/D150A/L151I are not associated with HL development neither in the simple and nor in the multivariable analyses.

**Table 1:** Comparison of LMP1 polymorphisms in blood from HIV-HL and from HIV-infected individuals without tumor.

Polymorphisms	Frequency	%†
**Sequences obtained from samples of 41 HIV-HL** *		
I124V/I152L	5	12.2
F144I/D150A/L151I	17	41.5
Other	20	48.8
**Sequences obtained from samples of 85 participants without tumor** §		
I124V/I152L	21	24.7
F144I/D150A/L151I	27	31.8
Other	41	48.2

*Single sequences were obtained from 31 participants and 2 sequences from 10 participants.

§ Single sequences were obtained from 36 participants, 2 sequences from 46 participants and 3 sequences from 3 participants.

† The total is more than 100% due to the simultaneous presence of both sets of polymorphisms in 1 HIV-HL and in 4 participants without tumor.

**Table 2:** Association between the presence of polymorphisms I124V/I152L or 144I/D150A/L151I and HIV-HL susceptibility measured by logistic regression.

Polymorphisms	OR	95% CI	OR*	95% CI*
I124V/I152L	0.42	[0.15/1.22]	0.44	[0.14/1.39]
F144I/D150A/L151I	1.52	[0.70/3.29]	1.25	[0.52/3.00]
I124V/I152L and 144I/D150A/L151I	0.98	[0.46/2.06]	0.78	[0.33/1.82]

*Adjusted for sex, age, number of years of antiretroviral therapy, CD4 levels, all at the date of blood sampling.

We further analyzed the distribution of the relevant polymorphisms in the corresponding biopsies from 16 HIV-HL (Figure 4B). LMP1 sequences were obtained from 15/16 biopsies, among them single sequences were obtained in biopsies from 10 participants and two sequences in biopsies from 5 participants. We found a good concordance between LMP1 sequences from HIV-HL biopsies and corresponding blood in 10/15 cases (Table 3). We also used EBV typing based on EBNA2 differences as a separate criteria to assess the correspondence between strains present in the blood and the biopsies as performed previously by Dolcetti et al. [30]. In this context, lack of correspondence by EBV typing between blood and biopsies would indicate that different EBV strains reside in the two compartments and that blood samples could not be used to monitor LMP1 polymorphisms. We were able to type EBV from the blood of 15 HIV-HL and from 12 biopsies and the data overall confirm the good concordance between blood and biopsy.

**Table 3:** Comparison of LMP1 polymorphisms and EBNA2 subtype in blood and in the corresponding biopsy of HIV-HL.

Cases		Sequences obtained			EBV subtype*
#	Sample	# LMP1	Polymorphisms	Identical sequences	
1	Blood	2	Others		1
	Biopsy	1	Other	Yes	NA
2	Blood	1	V124		1+2
	Biopsy	1	Other	No	1
3	Blood	1	I144		1
	Biopsy	1	I144	Yes	NA
4	Blood	1	Other		2
	Biopsy	1	Other	Yes	2
5	Blood	1	Other		1
	Biopsy	1	Other	Yes	1
6	Blood	1	I144		2
	Biopsy	2	I144 - Other	Yes	2
7	Blood	1	I144		2
	Biopsy	2	I144 - Other	Yes	1+2
8	Blood	1	Other		1+2
	Biopsy	1	Other	No	1+2
9	Blood	1	Other		1+2
	Biopsy	1	Other	Yes	1+2
10	Blood	2	I144		1+2
	Biopsy	1	I144	Yes	1
11	Blood	1	Other		1+2
	Biopsy	1	Other	No	1+2
12	Blood	1	Other		1+2
	Biopsy	2	V124 - I144	No	2
13	Blood	1	Other		NA
	Biopsy	0	NA	NA	NA
14	Blood	1	I144		1
	Biopsy	1	I144	Yes	1
15	Blood	1	I144		1
	Biopsy	2	Others	No	NA
16	Blood	1	Other		1
	Biopsy	1	Other	Yes	1

NA: not amplified.

*based on EBNA2 differences.

## Discussion

In the present study polymorphisms leading to enhanced LMP1-mediated NF-κB activation were identified and their importance in the etiology of EBV-associated HIV-HL was assessed in a pilot epidemiological study. Overall, the results presented here improve the understanding of the landscape of LMP1 genetic variation associated with NF-κB activation and show that markers of increased NF-κB activation levels *in vitro* are not predictive factors for EBV-associated HIV-HL susceptibility in the SHCS.

Sequence variations in LMP1 gene have been described in a Chinese NPC derived isolate [14] and isolates from various EBV-associated tumors. The 30 bp deletion in the C-terminus, which was first identified in LMP1 isolate CAO and subsequently in isolates from HL [14], [31], [32], was the most commonly reported LMP1 modification (reviewed in [33]). Genetic variations were extensively studied and led to several classification systems. The first was based on amino acid changes in the C-terminal domain relative to the prototypic B95-8 LMP1; the different classes were named according to the geographical region from which the initial isolate was derived [29], [34]. A second classification system defined 22 patterns based on amino acid changes in the C-terminal domain upstream and downstream the repeat region [35]. Finally, a last classification system defined four groups named A to D based on LMP1's full length sequence [36], [37]. One significant difference found among variants is their ability to activate NF-κB transcription factor. Induction of higher NF-κB activation levels by CAO LMP1 compared to B95-8 LMP1 was consistently observed in several cell lines of B cell and epithelial origin when measured by gene reporter assays [15], [16], [37], [38]. Increased NF-κB activation were also reported for variants Alaskan, China1 and Med+ [38], G50, G64 and NPC7 [37] and C15 [39]. However, the exact polymorphisms allowing increased NF-κB activation levels have not been mapped to date. In this study we characterized full length LMP1 variants from patients with EBV-associated lymphoma and from HIV-infected individuals enrolled in the SHCS. Our results showed that a number of variants induced higher NF-κB activation levels compared to B95-8 LMP1 by gene reporter assays. Some variants were highly homologous to CAO LMP1, but other variants were more distantly related based on a phylogenetic analysis. Using LMP1 chimera and targeted mutagenesis we identified single polymorphisms in natural LMP1 variants that are responsible for the enhanced NF-κB activation of these variants. Amino acid change F144I is present in variants that cluster with CAO in a phylogenetic analysis and that have the ability to activate NF-κB at high levels. This group of variants is more closely related to China1 than to the other LMP1 strains with F144I described by Edwards et *al.* [29], [38]. The linked polymorphisms _ENREF_33F144I/D150A/L151I are distinctive features of this group of variants. F144I was also reported in LMP1 variants isolated from several malignancies that do not carry the deletion, such as the European group C, and the subgroup Ch2 and AL [29], [37]. EBV type 2 LMP1 AG876 does not carry the mutation F144I but carry a 10 amino acid deletion indicating that F144I and the 10 amino acid deletion do not always segregate together. Most interestingly, we observed a high frequency of the polymorphism F144I in published sequences of LMP1 from EBV associated tumors: 5/5 variants isolated from Vietnamese patients with NPC [40], 21/21 variants isolated from patients with NPC in the southern China [41], 7/7 variants isolated from patients with nasal NK/T-cell lymphoma [42], 5/5 variants from NPC in Hong Kong [43], and in 10/12 variants isolated from salivary gland lymphoepithelial carcinoma [44]. Another amino acid change associated with enhanced NF-κB activation is I124V. This polymorphism always segregates with I152L and was not previously described. An additional amino acid change associated with enhanced NF-κB activation is F106Y. This polymorphism is present in all LMP1 variants isolated in our study. F106Y was shown to be present in the population at a high frequency. It is present in CAO LMP1 and LMP1 subgroups A, B, C and D [37] and in the subgroups Ch1, Ch2, AL and NC [29]. F106Y is also present in LMP1 AG876 [45]. We found that the introduction of mutation F106Y in B95-8 LMP1 led to enhanced NF-κB activation compared to wild type B95-8 LMP1. The back mutation Y106F in variants 7795, 7815, 7918, 7821 and 7948 led to a significant down modulation of NF-κB activation, demonstrating that F106Y is the polymorphism responsible for the increased signaling by these variants. However, since F106Y is also present in variants such as P1 that induced NF-κB activation levels close to B95-8 LMP1, the effect of this polymorphism on signaling is likely to be modulated by one or several polymorphisms yet to be mapped.

Our results extend the previous findings by Blake et al., showing a role of the transmembrane domain for regulating CAO LMP1's enhanced signaling activity [15]. We identified F144I as responsible for the enhanced NF-κB by variant A2, which is a close relative of CAO and showed that all the variants we isolated in our study that carry isoleucine at position 144 display the same phenotype.

CAO LMP1 not only show an increased ability to activate NF-κB [15], [16], [37], [38], but also a higher oncogenic potential compared to B95-8 LMP1 in a nude mice model by inducing phenotypic changes in a non-tumorigenic human keratinocyte line [46]. This led us to test whether polymorphisms responsible for increased LMP1-mediated NF-κB activation levels *in vitro* were associated with EBV-related cancers and more precisely with HIV-HL. The risk of HL in HIV-infected individuals is significantly higher than in the general population [22]. The use of HAART that improved the immunity status of HIV-infected individuals is associated with reduced incidence of Kaposi's sarcoma and high grade non-Hodgkin's lymphoma, but paradoxically with an increased risk of HIV-HL [47]. Moreover, HL risk could not be associated with absolute CD4^+^ cell counts [21]. Hodgkin's lymphoma arising in HIV-infected individuals show distinctive features compared to the HL cases in the general population, among them the association of HIV-HL with EBV in most cases [19], [20], [48]. The high frequency of EBV association with HIV-HL and the detection of a strong expression of LMP1 in tumor tissue in the context of a type II latency pattern [19], [49], [50] indicate that LMP1 is a relevant factor involved in the pathogenesis of this disease. Additional evidence highlights the central importance of the NF-κB signaling pathway for pathogenesis: NF-κB activation by LMP1 is critical for B cell transformation *in vitro* and *in vivo* [4], [13], [51], [52], [53], NF-κB hyperactivity was shown to be associated with tumorigenesis [54] and mutations in NF-κB regulatory pathways leading to increased activity have been identified in EBV-negative HL [55], [56], [57]. Polymorphisms I124V/I152L and F144I/D150A/L151I were chosen as markers of enhanced NF-κB activation in a pilot epidemiological study within the SHCS. Both sets of polymorphisms were found in EBV from blood of SHCS participants with or without HL diagnosis. We observed that I124V/I152L and F144I/D150A/L151I, combined, were found in virus from more than 50% of the participants from each group. Concordant with a high distribution in the two groups of patients, the two sets of polymorphisms are not identified as predictive factors associated with HL when analyzed by a logistic regression model, either together or individually.

More than one variant was detected in some samples, as demonstrated by EBV typing based on EBNA-2 gene and analysis of LMP1 sequences. The percentage of samples presenting a co-infection by EBV type 1 and type 2 was concordant with what was previously reported in the literature [58]. The presence of distinct EBV strains in the blood and tumor site has been previously reported [59], but another study showed a good concordance with respect to EBNA-2 region and LMP1 gene in normal and neoplasic cells [30]. In order to determine to which extend EBV strains differ between blood and tumor biopsy samples on this group of patients, we compared LMP1 sequences present in blood from HIV-HL and in its corresponding biopsy. EBV typing based on EBNA-2 was chosen as a separate marker. Based on LMP1 sequences, we found a good correspondence between blood and tumor samples in 10/15 cases, and by using EBV typing based on EBNA-2 we found a good correspondence between blood and tumor samples in 12/12 cases. The relatively good correspondence of LMP1 variants between blood and tumor biopsies indicates that larger future studies using blood samples would be relevant to the identification of viral polymorphisms influencing the development of EBV-associated malignancies. Overall our analysis showed no major difference in the profile of LMP1 polymorphisms in HIV-infected individuals without EBV-associated tumors, HIV-HL and other EBV-associated cancers. The characterization of full-length LMP1 sequences obtained from EBV-associated lymphoma and HIV-infected individuals in the SHCS revealed a high number of identical polymorphisms. Similar genetic variations were reported in other tumor types such as NPC. Our phylogenetic analysis confirmed the relationship among the various LMP1 isolates and interestingly the distribution of ethnic groups was not related to particular branches or to specific sets of polymorphisms.

Overall the pilot clinical study on the potential association of polymorphisms I124V/I152L and F144I/D150A/L151I with HL demonstrated the feasibility and interest of such an approach, even if the statistical power was too low to draw a definitive conclusion, which is a major limit of this study. A further step of this work would be the extension to a larger cohort and inclusion of a higher number of HL.

Taken together, our results showed that enhanced NF-κB activation resulted from single amino acid changes in the transmembrane segments 4 and 5 of the protein: F106Y, I124V and F144I. Moreover, they demonstrated that several groups of LMP1 variants, through distinct mutational paths, mediate enhanced NF-κB activation levels compared to B95-8 LMP1.

## Supporting Information

Figure S1 **Determination of the half-life of B95-8, A2 and P1 LMP1.** (A) Detection of B95-8 LMP1 in LCL and in transfected 293T cells. For the transfection: 293T cells were transfected with B95-8 LMP1 vector. B95-8 LCL cells were grown in RPMI. Both kinds of samples were lysed and the protein amount measure with BCA (Pierce). B95-8 LCL sample was serially diluted with H2O. Samples were then separated on a 10% SDS PAGE and immunobloted with S12 anti-LMP1 antibody. Comparable LMP1 expression levels were observed upon transfection and in LCL. (B–D) Determination of LMP1's half-life by pulse-chase labeling. The method used for the labeling is described in Materials and Methods S1. (B) Scan of the radioactive signals. (C) Plotted quantifications of the signals detected in (B) and determination of the half-life with a non-linear regression model using GraphPad Prism version 5.03 for Windows. (D) Histogram of the half-life and values.
(TIF)

Figure S2 **Alignment of LMP1 variants used in the study and specific GenBank accession number (BankIt1498710) for each new LMP1 sequence.**
(DOCX)

Figure S3 **NF-κB activation levels by LMP1 chimeras.** (A) Schematic representation of LMP1 chimeras split at both amino acids 118 and 231. The six transmembrane segments are represented by boxes. (B) NF-κB activation by LMP1 chimeras. HEK cells were transfected with 50 ng of LMP1 vector and 50 ng of NF-κB reporter plasmid. Empty vector was used as control. NF-κB activity was measured twenty-four hours after transfection using luciferase assay (Promega). Shown are representative of three independent experiments with similar results. Data are given as mean ± SD of triplicates. Statistical analysis was done using one-way ANOVA with Bonferroni posttest using GraphPad Prism. * P<0.05, **** P<0.0001 relatively to the NF-κB activation of B95-8 LMP1. RLU: relative light units.
(TIF)

Figure S4 **NF-κB activation.** (A–B) NF-κB activation by LMP1 mutants based on B95-8 background and on variants background. HEK cells were transfected with 50 ng of LMP1 vector and 50 ng of NF-κB reporter plasmid. Empty vector was used as control. NF-κB activity was measured twenty-four hours after transfection using luciferase assay (Promega). Shown are representative of three independent experiments with similar results. Data are given as mean ± SD of triplicates. Statistical analysis was done using one-way ANOVA with Bonferroni posttest using GraphPad Prism. ** P<0.01, *** P<0.001 relatively to the NF-κB activation of B95-8 LMP1. RLU: relative light units. (A) LMP1 mutants with positions 124 and 152 mutated in B95-8 and variant in 7825, a member of the first group of variants. (B) LMP1 mutants with position 106 mutated in B95-8 and in LMP1 variants of the third group.
(TIF)

Table S1 **NF-κB activation levels of LMP1 prototype and mutants.**
(DOCX)

Materials and Methods S1 Determination of protein half-life by pulse chase analysis. Primers and PCR conditions used for LMP1 amplification from genomic DNA. EBV typing.
(DOCX)
