# Supplementary figures and images for: Genetic Diversity of EBV-Encoded LMP1 in the Swiss HIV Cohort Study and Implication for NF-Κb Activation
Source: PLoS One. 2012 Feb 22;7(2):e32168. doi: 10.1371/journal.pone.0032168 (PMC3285206; doi:10.1371/journal.pone.0032168)

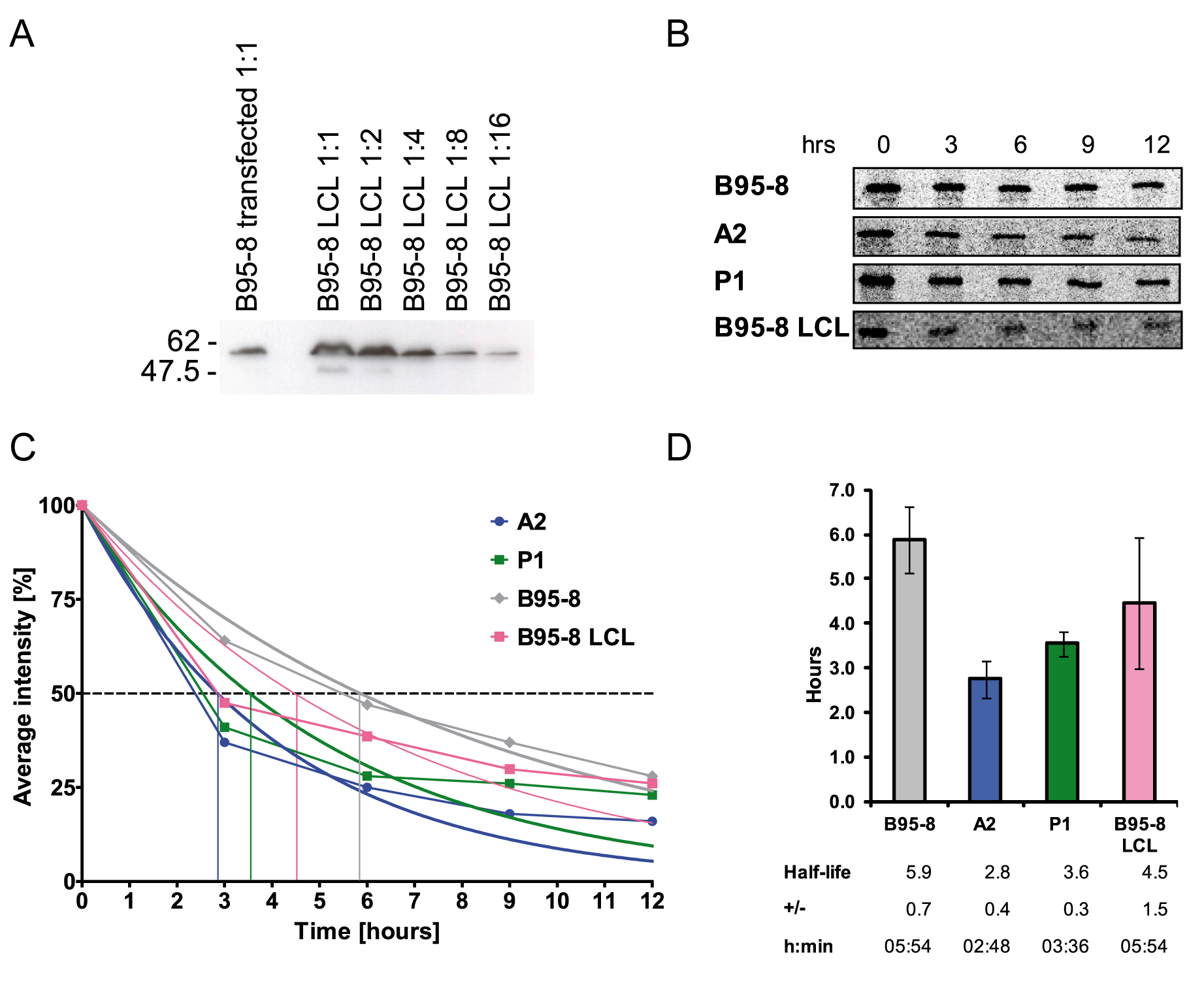

Supplement: Figure S1 — Determination of the half-life of B95-8, A2 and P1 LMP1. (A) Detection of B95-8 LMP1 in LCL and in transfected 293T cells. For the transfection: 293T cells were transfected with B95-8 LMP1 vector. B95-8 LCL cells were grown in RPMI. Both kinds of samples were lysed and the protein amount measure with BCA (Pierce). B95-8 LCL sample was serially diluted with H2O. Samples were then separated on a 10% SDS PAGE and immunobloted with S12 anti-LMP1 antibody. Comparable LMP1 expression levels were observed upon transfection and in LCL. (B–D) Determination of LMP1's half-life by pulse-chase labeling. The method used for the labeling is described in Materials and Methods S1. (B) Scan of the radioactive signals. (C) Plotted quantifications of the signals detected in (B) and determination of the half-life with a non-linear regression model using GraphPad Prism version 5.03 for Windows. (D) Histogram of the half-life and values. (TIF) [file pone.0032168.s001.tif]

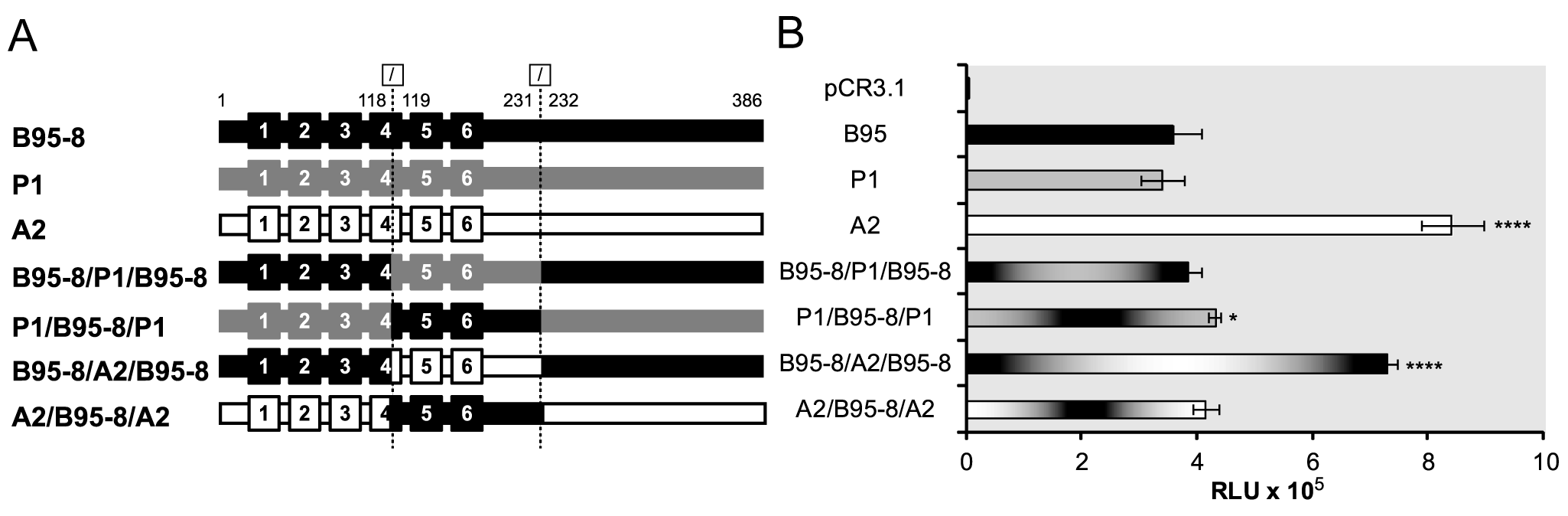

Supplement: Figure S3 — NF-κB activation levels by LMP1 chimeras. (A) Schematic representation of LMP1 chimeras split at both amino acids 118 and 231. The six transmembrane segments are represented by boxes. (B) NF-κB activation by LMP1 chimeras. HEK cells were transfected with 50 ng of LMP1 vector and 50 ng of NF-κB reporter plasmid. Empty vector was used as control. NF-κB activity was measured twenty-four hours after transfection using luciferase assay (Promega). Shown are representative of three independent experiments with similar results. Data are given as mean ± SD of triplicates. Statistical analysis was done using one-way ANOVA with Bonferroni posttest using GraphPad Prism. * P<0.05, **** P<0.0001 relatively to the NF-κB activation of B95-8 LMP1. RLU: relative light units. (TIF) [file pone.0032168.s003.tif]

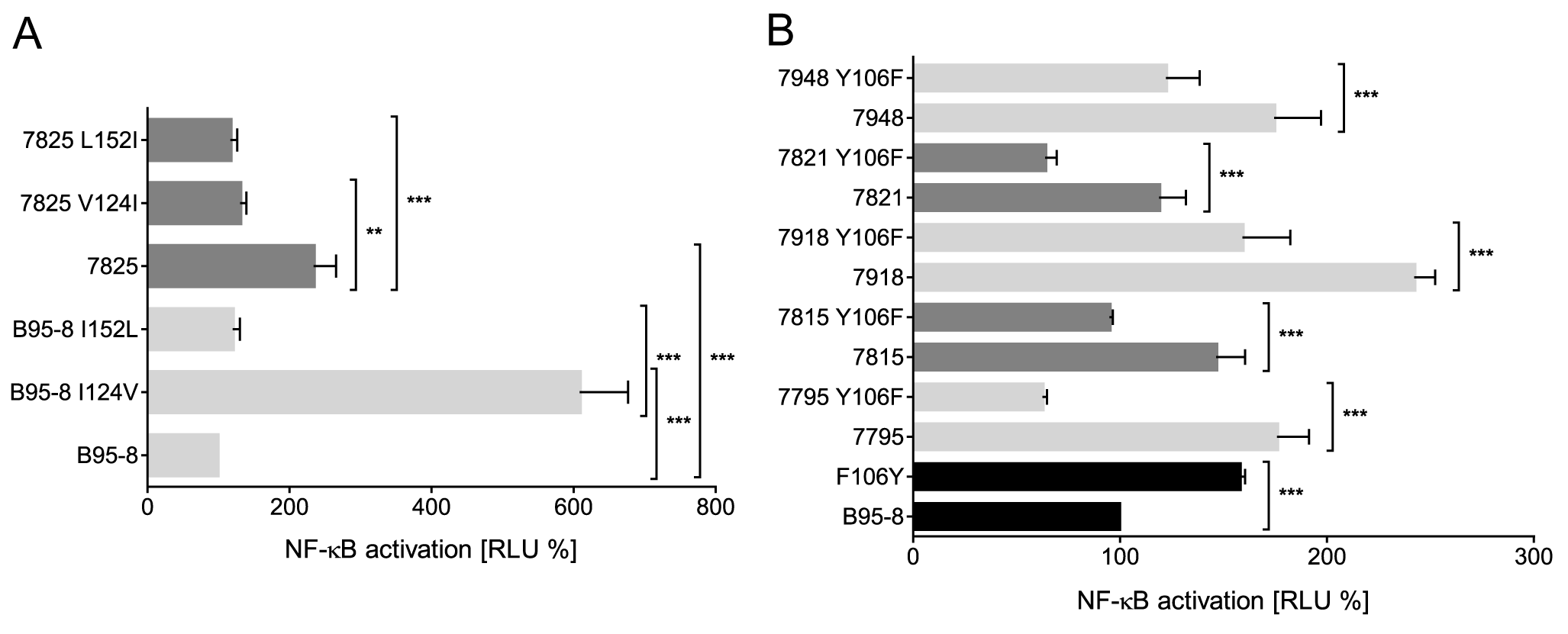

Supplement: Figure S4 — NF-κB activation. (A–B) NF-κB activation by LMP1 mutants based on B95-8 background and on variants background. HEK cells were transfected with 50 ng of LMP1 vector and 50 ng of NF-κB reporter plasmid. Empty vector was used as control. NF-κB activity was measured twenty-four hours after transfection using luciferase assay (Promega). Shown are representative of three independent experiments with similar results. Data are given as mean ± SD of triplicates. Statistical analysis was done using one-way ANOVA with Bonferroni posttest using GraphPad Prism. ** P<0.01, *** P<0.001 relatively to the NF-κB activation of B95-8 LMP1. RLU: relative light units. (A) LMP1 mutants with positions 124 and 152 mutated in B95-8 and variant in 7825, a member of the first group of variants. (B) LMP1 mutants with position 106 mutated in B95-8 and in LMP1 variants of the third group. (TIF) [file pone.0032168.s004.tif]
