# Supplementary material for: Genetic Diversity of EBV-Encoded LMP1 in the Swiss HIV Cohort Study and Implication for NF-Κb Activation
Source: PLoS One. 2012 Feb 22;7(2):e32168. doi: 10.1371/journal.pone.0032168 (PMC3285206; doi:10.1371/journal.pone.0032168)
Supplement: Figure S2 — Alignment of LMP1 variants used in the study and specific GenBank accession number (BankIt1498710) for each new LMP1 sequence. (DOCX) [file pone.0032168.s002.docx]

**Figure S2**

10 20 30 40 50 60 70 80 90 100

....|....|....|....|....|....|....|....|....|....|....|....|....|....|....|....|....|....|....|....|

**Raji**  **.DL.....................I.................I..N..........A.....V.....................L...............**

**7836**  **.DL.....................I.................I..N..........A.....L.....................L...............**

**7885**  **.DL.....................I.................I..N..........A.....V.....................L...............**

**7850**  **.DL.....................I.................I..N..........A.....V.....................L...............**

**7825**  **.DL.....................I.................I..N..........A.....V.....................L...............**

**7939**  **.DL.....................I.................I..N..........A.....V.....................L...............**

**7924**  **.--................................I.........N...............................Y...G.GL...............**

**A2**  **..R.........P...L.......I...V.............L..N.....................L.............G.GL...............**

**CAO**  **..R.........P...L.......I........................................................G.GL...............**

**7705**  **.............................................N...................................G.GL...............**

**7950**  **......................F......................NL.A................................G.GL...............**

**7823**  **.................................F...........N...................................G.GL...............**

**7910**  **................Q............................N...................................G.GL...............**

**7880**  **.DR.....................IT.......V........I.............A...I.................T...IAL...............**

**7859**  **.DL.................................................................................L...............**

**B95-8**  **MEHDLERGPPGPRRPPRGPPLSSSLGLALLLLLLALLFWLYIVMSDWTGGALLVLYSFALMLIIIILIIFIFRRDLLCPLGALCILLLMITLLLIALWNL**

**7848**  **.......................F...........I............................L...................L...............**

**7835**  **....................................................................................L...............**

**7881**  **.............................................N......................................L...............**

**7902**  **...........................V........................................................L...............**

**AG876**  **............................................................I.......................L...............**

**P1**  **.............................................H.........................L............L...............**

**7769**  **.............................................N......................................L...............**

**7926**  **.............................................N................L.....................L...............**

**7795**  **..........................................................G.........................L...............**

**7886**  **....................................................................................L...............**

**A3**  **...................................................................L................L...............**

**7918**  **..................................V.................................................L...............**

**7948**  **...................................................................L................L...............**

**7843**  **....................................................................................L...............**

**7815**  **............................................................I.......................L...............**

**A1**  **..............................................................L.....................L...............**

**7703**  **....................................................................................L...............**

**7914**  **.................R..................................................................L...............**

**7798**  **...................................................................L................L...............**

**7913**  **...................................................................L................L...............**

**7849**  **...................................................................L................L..........V....**

**7821**  **...................................................................L................L...............**

**7893**  **.................................S.................................L................L...............**

110 120 130 140 150 160 170 180 190 200

....|....|....|....|....|....|....|....|....|....|....|....|....|....|....|....|....|....|....|....|

**Raji**  **.....Y...............L......I.....................I.................................................**

**7836**  **Q....Y.................V.F..I.....................IL................................................**

**7885**  **.....Y.................V.F..I.....................IL................................................**

**7850**  **.....Y.................V.F..I.....................IL................................................**

**7825**  **.....Y.................V.F..I.....................IL................................................**

**7939**  **.....Y.................V.F..I.....................IL..........................V.....................**

**7924**  **.....Y...............L...F..I..............I.....AI.....................................P..T........**

**A2**  **.....Y...............L...F..I..............I.....AI.....................................P..T........**

**CAO**  **.....Y...............L...F..I.......L......I.....AI..........................M..........P..T........**

**7705**  **.....Y...............L...F..I..G...........I.....AI.....................................P..T........**

**7950**  **.....Y...............L...F..I..G...........I.....AI.....................................P..T........**

**7823**  **.....Y...............L...F..I..G...........I.....AI.....................................P..T........**

**7910**  **.....Y...............L...F..I..G...........I.....AI.....................................P..T........**

**7880**  **.....Y...............L....M.I.....................I............................L....................**

**7859**  **R....Y..............................................................................................**

**B95-8**  **HGQALFLGIVLFIFGCLLVLGIWIYLLEMLWRLGATIWQLLAFFLAFFLDLILLIIALYLQQNWWTLLVDLLWLLLFLAILIWMYYHGQRHSDEHHHDDS**

**7848**  **.....Y..............................................................................................**

**7835**  **.....Y..............................................................................................**

**7881**  **.....Y..............................................................................................**

**7902**  **.....Y...............V...........D..V...............................................................**

**AG876**  **.....Y...............L......I.......................................................................**

**P1**  **.....Y...............L..............................................................................**

**7769**  **.....Y...............L......I.......................................................................**

**7926**  **.....Y...............L......I.......................................................................**

**7795**  **.....Y...............L......I.......................................................................**

**7886**  **N....Y......................I.....................I...........H............................T........**

**A3**  **.....Y...............L......I.......................................................................**

**7918**  **.....Y...............L......I.......................................................................**

**7948**  **.....Y...............L...F..I.......L...............................................................**

**7843**  **.....Y...............L......I.......................................................................**

**7815**  **.....Y...............L...F..I.......................................................................**

**A1**  **.....Y...............L......I.......................................................................**

**7703**  **.....Y...............L......I.......................................................................**

**7914**  **.....Y...............L......I.......................................................................**

**7798**  **.....Y...............L..............................................................................**

**7913**  **.....Y...............L......I.......................................................................**

**7849**  **.....Y...............L......I.......................................................................**

**7821**  **.....Y...............L......I.......................................................................**

**7893**  **.....Y...............L......I.......................................................................**

210 220 230 240 250 260 270 280 290 300

....|....|....|....|....|....|....|....|....|....|....|....|....|....|....|....|....|....|....|....|

**Raji**  **...........SNQ..........L........................N.........................-----------..............**

**7836**  **...........SNQ.............................................................QDPDNTDDNGP..............**

**7885**  **...........SNQ..........Y..................................................QDPDNTDDNGP...........N..**

**7850**  **...........SNQ.............................................................QDPDNTDDNGP..............**

**7825**  **...........SNQ.............................................................QDPDNTDDNGP..............**

**7939**  **.........A.SNQ.............................................................QDPDNTDDNGP..............**

**7924**  **...........S....................................................R..........QDPDNTDDNGP-----.........**

**A2**  **...........S...............................................................QDPDNTDDNGP-----.........**

**CAO**  **...........S...............................................................QDPDNTDDNGP-----.........**

**7705**  **...........S................T..............................................QDPDNTDDNGP-----.........**

**7950**  **...........S...............................................................QDPDNTDDNGP-----.........**

**7823**  **...........S...............................................................QDPDNTDDNGP-----.........**

**7910**  **...........S........D......................................................QDPDNTDDNGP-----.........**

**7880**  **...........S................T......................A.......................QDPDNTDDNGP-----.G.......**

**7859**  **...........S...............................................................QDPDNTDDNGP-----.........**

**B95-8**  **LPHPQQATDDSGHESDSNSNEGRHHLLVSGAGDGPPLCSQNLGAPGGGPDNGPQDPDNTDDNGPQDPDNTDDNGP-----------HDPLPQDPDNTDDN**

**7848**  **...........S...............................................................-----------..............**

**7835**  **...........S............R..................................................-----------..............**

**7881**  **........H..S.L.............................................................QDPDNTDDNGP..............**

**7902**  **........H..SN............................................D.................-----------............N.**

**AG876**  **............................T..............................................QDPDNTDDNGP-----......A..**

**P1**  **............................T......................A.......................QDPDNTDDNGP-----.G.......**

**7769**  **.............Q........S.....T......................A.......................QGPDNTDDNGP-----.G.......**

**7926**  **........H..A................T......................A.............G.........QGPDNTDDNGP-----.G.......**

**7795**  **...........S................T......................A.......................QGPDNTDDNGP-----.G.......**

**7886**  **...........S................T......................A.......................QGPDNTDDNGP-----.G.......**

**A3**  **...........S................T..............................................QGPDNTDDNGP-----.G.......**

**7918**  **...........S................T....................................G.........QGPDNTDDNGP-----.G.......**

**7948**  **...........S................N......................A.............G.........QGPDNTDDNGP-----.G.......**

**7843**  **...........SR...............T......................A.......................QGPDNTDDNGP--------------**

**7815**  **...........S................T......................A.......................QDPDNTDDNGP-----.........**

**A1**  **...........S............R...T......................A.......................QDPDNTDDNGP-----.........**

**7703**  **...........S.Q.......A......T......................A.......................QGPDNTDDNGP-----.........**

**7914**  **...........S.Q..............T..............................................QDPDNTDDNGP-----.G.......**

**7798**  **...........S.Q..............T......................A.......................QDPDNTDDNGP-----.........**

**7913**  **...........S.Q..............T......................A.............G.........QGPDNTDDNGP-----.G.......**

**7849**  **...........S................P......................A.......................QDPDNTDDNGP-----.G.......**

**7821**  **...........S...........R....T......................A.......................QDPDNTDDNGP-----.........**

**7893**  **...........S.......D...R....T......................A.......................QDPDNTDDNGP-----.G......D**

310 320 330 340 350 360 370 380 390 400

....|....|....|....|....|....|....|....|....|....|....|....|....|....|....|....|....|....|....|....|

**Raji**  **.............----------------------......N...........................................D.I..........T.**

**7836**  **.S...........----------------------......N............N...........R...S.......----------..........T.**

**7885**  **.............----------------------......N............N...........R...S.......----------..........T.**

**7850**  **.............----------------------......N............N...........R...S.......----------..........T.**

**7825**  **.............QDPDNTDDNGP-----------......N........A...N...........R...S.......----------..........T.**

**7939**  **.............----------------------......N............N...........R...S.......----------..........T.**

**7924**  **.............----------------------......N............N...........R...S.......----------..........T.**

**A2**  **.............----------------------......N............N...........R...S.......----------..........T.**

**CAO**  **.............QDPDNTDDNGPQDPDNTDDNGP......N............N.....A.....R...S.......----------..........T.**

**7705**  **..---------------------------------......N............NF..........R...S.......----------..........T.**

**7950**  **..---------------------------------......N............N.......T...R...S.......----------..........T.**

**7823**  **.............----------------------Q.....N............N...........R...S.......----------..........T.**

**7910**  **.............QDPDNTDDNGP-----------......N............N..........HR...SV......----------..........T.**

**7880**  **..---------------------------------......N............E...........R...S.............R.............T.**

**7859**  **.............----------------------......N............N...........R...S.......----------..........T.**

**B95-8**  **GPQDPDNTDDNGP----------------------HDPLPHSPSDSAGNDGGPPQLTEEVENKGGDQGPPLMTDGGGGHSHDSGHGGGDPHLPTLLLGSS**

**7848**  **.............----------------------........N...............................................I......T.**

**7835**  **.............----------------------.........................Q.......................N....S........T.**

**7881**  **.............----------------------.........................Q......................S..............T.**

**7902**  **.............----------------------.........................Q.....................................T.**

**AG876**  **..---------------------------------......N............N...........R...S.......----------..........T.**

**P1**  **...G.........----------------------......N............E...........R...S...........................T.**

**7769**  **...G.........QGPDNTDDNGP-----------......N.......E....E..........-----------------------......M...A.**

**7926**  **...G.........----------------------......N.......E.R..E..........-----------------------..........A.**

**7795**  **..---------------------------------......N............E...........R...S.............R.............T.**

**7886**  **..---------------------------------......N............E...........R...S.............R.............T.**

**A3**  **..---------------------------------......N............E...........R.................R.............T.**

**7918**  **...G.........----------------------......D............E...........R...S.............R.............T.**

**7948**  **...G.........QGPDNTDDNGPQGPDNTDDNGP......N............E.....Q.....R...S............RR.............T.**

**7843**  **-----------------------------------......N............D...........R...S.......----------..........T.**

**7815**  **..---------------------------------......N............E...........R...S..........A..R.............T.**

**A1**  **.............QGPDNTDDNGP-----------......N............E...........R...S.............R.............T.**

**7703**  **...G.........QGPDNTDDNGP-----------......N............E...........R...S.............R.............T.**

**7914**  **...G.........----------------------......N.......N....E...........R...S..........G..R.............T.**

**7798**  **...G.........QGPDNTDDNGPQGPDNTDDNGP......N.......Y.H..E...........R...S.............R..C..........T.**

**7913**  **..---------------------------------......K.G.....Y....E.......T...R...S.......----------..........T.**

**7849**  **...G.........----------------------......N.......N.D.............AR...S.............R..C..........T.**

**7821**  **...G.........QGPDNTDDNGP-----------......N.......N.D..............R...S.............R..C..........T.**

**7893**  **..---------------------------------......N.......N.D.............HR..SS.............R..C..........T.**

410 420

....|....|....|....|

**Raji**  **...................***

**7836 ...................* 7836 JQ240434**

**7885 ...................* 7885 JQ240435**

**7850 ...................* 7850 JQ240436**

**7825 ...................* 7825 JQ240437**

**7939 ...................* 7939 JQ240438**

**7924 ...................* 7924 JQ240439**

**A2 ...................* A2   JQ240440**

**CAO ...................***

**7705 ...................* 7705 JQ240441**

**7950 ...................* 7950 JQ240442**

**7823 ...................* 7823 JQ240443**

**7910 ...................* 7910 JQ240444**

**7880 ...................* 7880 JQ240445**

**7859 ...................* 7859 JQ240446**

**B95-8 GSGGDDDDPHGPVQLSYYD***

**7848 ...................* 7848 JQ240447**

**7835 ...................* 7835 JQ240448**

**7881 ...................* 7881 JQ240449**

**7902 ...................* 7902 JQ240450**

**AG876**  **...................***

**P1**  **...................*** **P1   JQ240451**

**7769**  **...................*** **7769 JQ240452**

**7926**  **...................*** **7926 JQ240453**

**7795**  **...................*** **7795 JQ240454**

**7886**  **...................*** **7886 JQ240455**

**A3**  **...................*** **A3   JQ240456**

**7918**  **...................*** **7918 JQ240457**

**7948**  **...................*** **7948 JQ240458**

**7843**  **...................*** **7843 JQ240459**

**7815**  **...................*** **7815 JQ240460**

**A1**  **...................*** **A1   JQ240461**

**7703**  **...................*** **7703 JQ240462**

**7914**  **...................*** **7914 JQ240463**

**7798**  **...................*** **7798 JQ240464**

**7913**  **...................*** **7913 JQ240465**

**7849**  **...................*** **7849 JQ240466**

**7821**  **...................*** **7821 JQ240467**

**7893**  **...................*** **7893 JQ240468**
