# Supplementary material for: Genetic Diversity of EBV-Encoded LMP1 in the Swiss HIV Cohort Study and Implication for NF-Κb Activation
Source: PLoS One. 2012 Feb 22;7(2):e32168. doi: 10.1371/journal.pone.0032168 (PMC3285206; doi:10.1371/journal.pone.0032168)
Supplement: Table S1 — NF-κB activation levels of LMP1 prototype and mutants. (DOCX) [file pone.0032168.s005.docx]

Table S1:

NF-κB activation levels of LMP1 prototype and mutants.

| LMP1 | | | % | | SD % | | p-value | |
| --- | --- | --- | --- | --- | --- | --- | --- | --- |
| Prototype | B95-8 | 100 | | 0 | |  | |  |
|  | B95-8 I85L | 101 | | 48 | | ns | |  |
|  | B95-8 F106Y | 296 | | 114 | | *** | |  |
|  | B95-8 L126F | 93 | | 35 | | ns | |  |
|  | B95-8 M129I | 76 | | 24 | | ns | |  |
|  | B95-8 F144I | 308 | | 108 | | *** | |  |
|  | B95-8 D150A | 83 | | 12 | | ns | |  |
| Mutants | B95-8 L151I | 64 | | 22 | | ns | |  |
|  | B95-8 Q189P | 87 | | 15 | | ns | |  |
|  | B95-8 S192T | 115 | | 23 | | ns | |  |
|  | B95-8 G212S | 87 | | 41 | | ns | |  |
|  | B95-8 F106Y | 289 | | 38 | | *** | |  |
|  | B95-8 F144I | 334 | | 30 | | *** | |  |
|  | A2 Y106F | 448 | | 60 | | *** | |  |
|  | A2 I144F | 167 | | 10 | | ns | |  |

NF-κB activities were measured 24 hours after transfection. Values are percentages ± SD of means of 9 independent transfections normalized to the activity of B95-8 LMP1 fixed at 100%. Significant p-values compared to the activation of B95-8 LMP1 are indicated with asterisks (*** p<0.001, one-way ANOVA with Bonferroni posttest performed using GraphPad Prism version 5.03 for Windows, GraphPad Software, San Diego California USA, www.graphpad.com).
