# Supplementary material for: Genetic Diversity of EBV-Encoded LMP1 in the Swiss HIV Cohort Study and Implication for NF-Κb Activation
Source: PLoS One. 2012 Feb 22;7(2):e32168. doi: 10.1371/journal.pone.0032168 (PMC3285206; doi:10.1371/journal.pone.0032168)
Supplement: Materials and Methods S1 — Determination of protein half-life by pulse chase analysis. Primers and PCR conditions used for LMP1 amplification from genomic DNA. EBV typing. (DOCX) [file pone.0032168.s006.docx]

**Supplementary material and methods**

### Determination of protein half-life by pulse chase analysis

HEK cells were transfected in 100-mm plates with 2 μg of the expression plasmids using Fugene 6. 24 hours post-transfection cells pooled from two 100-mm plates were seeded in six 60-mm plates coated with poly-L-Lysine and grown for 14 hours. Cells were starved at 37°C for 30 minutes in methionine and cysteine-free DMEM supplemented with 2% FCS and pulse-labeled with 100 μCi [^35^S] labeled cysteine/methionine per ml (Hartman) at 37°C for 45 minutes. Cells were washed with complete medium and then replaced in the incubator with complete DMEM for incubation from 0 to 12 hours chase times. Cells were rinsed with chilled PBS and lysed in RIPA buffer (150 mM NaCl, 20 mM Tris-HCl pH 8, 0.5% sodium deoxycholate, 1% Triton X-100, 0.1% SDS) supplemented with inhibitors of proteases (minicomplete, Roche Applied Science) and inhibitors of phosphatases (20 mM NaF, 25 mM glycerophosphate, and 1 mM orthovanadate). Lysates were cleared by centrifugation at 16,000g for 10 minutes and deep frozen in nitrogen at each time point. Samples were immunoprecipitated with mouse anti-LMP1 S12 monoclonal antibody bound to protein-A sepharose for 2 hours at 4°C. Precipitates were washed three times with RIPA buffer and twice with phosphate buffered saline and boiled for 4 min in 50 μl of 2x sample buffer and separated by 10 % SDS-PAGE. Gels were dried, exposed on a phosphor screen cassette and then the signal was read with a Typhoon (Amersham Biosciences) and quantification was done using ImageQuantTM TL2005 software (Amersham Biosciences).

### Primers and PCR conditions used for LMP1 amplification from genomic DNA

The region between positions 169.508 and 168.111 of EBV genome was amplified using a pair of primers based on the published prototype B95-8 LMP1 sequence [[1](#_ENREF_1)]:

Fwd: 5’-TCAACTGCCTTGCTCCTGACAC-3’

Rev: 5’-aggcaagcctatgacatggtaatgc-3’.

PCRs were carried out either with Pwo DNA Polymerase (Roche Applied Science): [98°C 5 min, 5 cycles (94°C 1 min, 65°C 1 min, 72°C 1.5 min), 5 cycles (94°C 1 min, 63°C 1 min, 72°C 1.5 min), 25 cycles (94°C 1 min, 60°C 1 min, 72°C 1.5 min), 5 cycles (94°C 1 min, 57°C 1 min, 72°C 1.5 min), 72°C 10 min] or with AmpliTaq Gold DNA Polymerase (Applied Biosystem): [95°C 10 min, 5 cycles (95°C 1 min, 65°C 1min, 72°C 1.5 min), 5 cycles (94°C 1 min, 63°C 1min, 72°C 1.5 min), 25 cycles (95°C 1 min, 60°C 1min, 72°C 2 min), 5 cycles (95°C 1 min, 57°C 1 min, 72°C 1.5min), 72°C 10 min] on the four biopsy samples used for the mapping of the polymorphisms and samples from HIV-infected individuals, respectively.

### EBV typing

EBV type was determined on EBNA2 gene by performing PCR and nested PCR on this gene with AmpliTaq Gold® DNA Polymerase (Applied Biosystems, Rotkreuz, Switzerland). Primers and PCR program used in our study are based on the publication of Telenti et al. [[2](#_ENREF_2)]:

Common forward primer: 5’-AGGGATGCCTGGACACAAGA-3’

Common reverse primer: 5’-TGGTGCTGCTGGTGGTGGCAAT-3’

EBV-1 forward nested primer: 5’-TCTTGATAGGGATCCGCTAGGATA-3’

EBV-1 reverse nested primer: 5’-ACCGTGGTTCTGGACTATCTGGATC-3’

EBV-2 forward nested primer: 5’-CATGGTAGCCTTAGGACATA-3’

EBV-2 reverse nested primer: 5’-AGACTTAGTTGATGCCCTAG-3’ PCR were carried out with this program: [94°C 10 min, 30 cycles (94°C 1.5 min, 60°C 1 min, 72°C 2 min), 72°C 10 min].

1. Fennewald S, van Santen V, Kieff E (1984) Nucleotide sequence of an mRNA transcribed in latent growth-transforming virus infection indicates that it may encode a membrane protein. J Virol 51: 411-419.

2. Telenti A, Uehlinger DE, Marchesi F, Germann D, Malinverni R, et al. (1993) Epstein-Barr virus infection in HIV-positive patients. Eur J Clin Microbiol Infect Dis 12: 601-609.
